# The Polysaccharides from *Codonopsis pilosula* Modulates the Immunity and Intestinal Microbiota of Cyclophosphamide-Treated Immunosuppressed Mice

**DOI:** 10.3390/molecules23071801

**Published:** 2018-07-20

**Authors:** Yu-Ping Fu, Bin Feng, Zhong-Kai Zhu, Xin Feng, Shu-Fan Chen, Li-Xia Li, Zhong-Qiong Yin, Chao Huang, Xing-Fu Chen, Bing-Zhao Zhang, Ren-Yong Jia, Xu Song, Cheng Lv, Gui-Zhou Yue, Gang Ye, Xiao-Xia Liang, Chang-Liang He, Li-Zi Yin, Yuan-Feng Zou

**Affiliations:** 1 Natural Medicine Research Center, College of Veterinary Medicine, Sichuan Agricultural University, Chengdu 611130, China; yupingfu424@163.com (Y.-P.F.); zhongkaizhu6@163.com (Z.-K.Z.); Xinfeng_felix@163.com (X.F.); chenshufan94@163.com (S.-F.C.); lilixia905@163.com (L.-X.L.); yinzhongq@163.com (Z.-Q.Y.); songx@sicau.edu.cn (X.S.); lvcheng1980@163.com (C.L.); yegang800206@163.com (G.Y.); liangxiaoxia@sicau.edu.cn (X.-X.L.); lorri190@126.com (C.-L.H.); yinlizi@hotmail.com (L.-Z.Y.); 2 Key Laboratory of Animal Disease and Human Health of Sichuan Province, College of Veterinary Medicine, Sichuan Agricultural University, Chengdu 611130, China; huangchao@sicau.edu.cn (C.H.); jiary@sicau.edu.cn (R.-Y.J.); 3 Animal Nutrition Institute, Sichuan Agricultural University, Chengdu 611130, China; fengbin@sicau.edu.cn; 4 Key Laboratory of Crop Ecophysiology and Farming System in Southwest China, Ministry of Agriculture, College of Agronomy, Sichuan Agricultural University, Chengdu 611130, China; chenxf64@sohu.com; 5 Shenzhen Institutes of Advanced Technology, Chinese Academy of Science, Shenzhen 518055, China; bz.zhang@siat.ac.cn; 6 Department of Applied Chemistry, College of Science, Sichuan Agricultural University, Chengdu 611130, China; yueguizhou@sicau.edu.cn

**Keywords:** *Codonopsis pilosula*, polysaccharides, intestinal microbiota, mucosal immunity, Immunosuppression

## Abstract

Based on previous studies about microflora regulation and immunity enhancement activities of polysaccharides from *Codonopsis pilosula* Nannf. var. *modesta* (Nannf.) L. T. Shen (CPP), there is little study on intestinal mucosal immunity, which is a possible medium for contacting microflora and immunity. In the present study, the BALB/c mice were divided into five groups (eight mice in each group), including a normal group (Con), a model control group (Model), and model groups that were administered CPP (50, 100, 200 mg/kg/d) orally each day for seven days after intraperitoneal injection of 60 mg/kg BW/d cyclophosphamide (CP) for three days. CPP recovered the spleen index and restored the levels of IFN-γ, IL-2, IL-10, as well as serum IgG. In addition, it elevated ileum secretory immunoglobulin A (sIgA), the number of *Lactobacillus* and acetic acid content in cecum. These results indicated that CPP plays an important role in the protection against immunosuppression, especially mucosa immune damage, and the inhibition of pathogenic bacteria colonization, which could be considered a potential natural source of immunoregulator.

## 1. Introduction

Cyclophosphamide (CP), a cytotoxic alkylating agent with a broad spectrum of activity, is widely used as an essential medicine in cancer treatment, blood and marrow transplantation (BMT). It could be converted to 4-hydroxycyclophosphamide, binding to DNA, and inducing apoptosis in immune cells [1]. It is used as a potential immunosuppressive agent (including innate immunity and adaptive immunity) as well, for auto-immune treatment [2] and immunosuppression animals model, which was manifested as lower activities of splenocytes, natural killer cell, macrophages and the reduction of immunomodulation-related cytokines and antibodies in serum, such as TNF-α, IFN-γ, IL-1α/β, IL-2, IgG, IgM, etc. [2,3,4]. At the same time, CP could disrupt the intestinal mucosal immunity by shortening the small intestinal villi, destroying the epithelial barrier, and reducing the expression of tight and adherens proteins, which may increase intestinal permeability and colonization resistance of pathogenic bacteria, depending on the change of microbial compositions in the gut [5,6,7]. However, the homeostasis of intestinal microbiota plays a key role in the adaptive and innate immune systems, through intestinal mucosa and epithelial cells [8], which activate the proliferation or differentiation of B/T lymphocyte cells, and the inflammation signal pathways [9,10,11]. The metabolites from undigested complex carbohydrates by bacterial fermentation in the colon, such as short-chain fatty acids (SCFAs), are essential energy sources and an immunoregulatory substance [12,13]. The damage of intestinal mucosa and dysbacteriosis may lead to the enhancement of pathogenic bacteria colonization, lower microbiota diversity, especially the reduction of the bacteria of the *Firmicutes* phylum, *Bacteroidetes* phylum (*Bacillus*, *Lactobacillus*, *Lactococcus*, etc.), and an increase in the *Proteobacteria* phylum, which was similar with the microbiota of inflammatory bowel diseases (IBD) patients [5,6,14,15,16].

Traditional Chinese Medicine restored the immunity injury by CP in much research, for instance, the plant extracts of *Picrorrhiza kurroa* (Scrophulariaceae), *Cassia occidentails* L. (Caesalpiniaceae), *Curculigo orchioides* Gaertn. (Amaryllidaceae) and their active ingredients such as polysaccharides, protein solutions, saponins, triterpenoids, phenolic compounds [1,8]. Radix Codonopsis is the root of *Codonopsis pilosula* (Franch) Nannf, *C. pilosula* Nannf. var. *modesta* (Nannf.) L. T. Shen, and *C. tangshen* Oliv, with good efficacies in lowering blood pressure, enhancing immunity, protecting blood vessels, improving microcirculation and strengthening the hematopoietic function, known as “poor man’s ginseng (*Panax ginseng*)” [17,18,19,20]. It is also used as health-care food, such as tonic wine and drinks mix [21]. *C. pilosula* has great variety of nutritional ingredients, such as polysaccharides, sterol, alkaloid, triterpenoids and essential oils, etc. [22]. The polysaccharides of *C. pilosula* has many biological activities, including facilitating the growth of *Bifidobacteri*, *Lactobacillus* [23,24], intestinal mucositis inhibition [23,25], tumor growth prevention [26,27], immune system modulation, and kidney protection [28], as well the activity of anti-senility [20,29] and anti-oxidant [30].

The polysaccharide from *C. pilosula* mentioned above is a well-known immunoregulatory substance, but with unclear active mechanism. In our previous study, the polysaccharide was isolated from *C. pilosula* Nannf. var. *modesta* L.T. Shen (CPP), and the purified neutral and pectic polysaccharides have shown prebiotic and complement fixing activity in vitro, respectively [31,32]. In addition, a pre-experiment of CPP in normal mice showed the activities of intestinal microbiota modulation and sIgA stimulation. Combing the complex structure and indigestible features in the upper digestive tract of polysaccharide, it was probable that the target organ of CPP was the intestine, through intestinal microbiota modulation and mucosal immunity enhancement. Consequently, this study was designed to investigate the protection activity of CPP on immunosuppressed mice, expounding and verifying the effect on immunological enhancement and intestinal flora.

## 2. Results

### 2.1. Isolation and Compositions Determination of Polysaccharides from C. pilosula

The crude water-soluble polysaccharides from *C. pilosula* were separated by precipitation with ethanol, and then lyophilized, named CPP (20%, *m*/*m*). The total polysaccharides, polyphenol and protein content were 89.30%, 0.35% and 0.63%, respectively.

### 2.2. Effects of CPP on Spleen, Thymus and Liver Index in CP-Treated Mice

**Figure 1:**
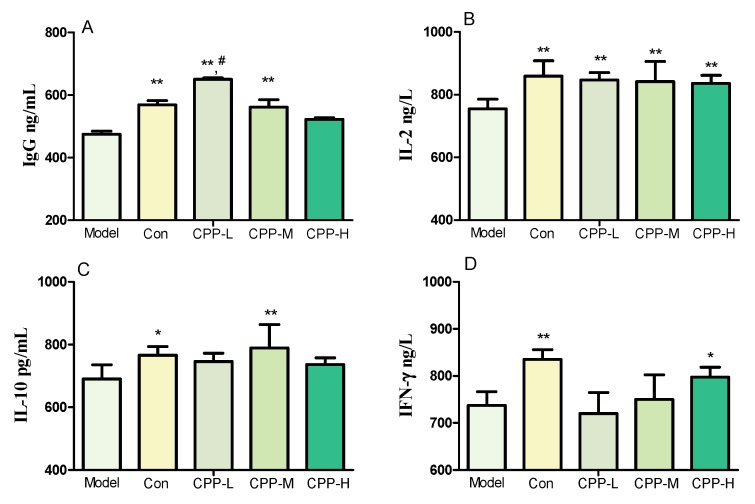
Effects of CPP on serum IgG (**A**), IL-2 (**B**), IL-10 (**C**), IFN-γ (**D**) concentration in cyclophosphamide-treated mice. Model, model control; Con, normal control; CPP-L, 50 mg/kg bodyweight CPP treated group; CPP-M, 100 mg/kg bodyweight CPP treated group; CPP-H, 200 mg/kg bodyweight CPP treated group. The values were presented as mean ± SD. * *p* < 0.05, compared with model group; ** *p* < 0.01, compared with model group; ^#^ *p* < 0.05, compared with normal group.

The essential immune function was reflected by the spleen, thymus and liver index. A dramatic decrease of spleen index (Table 1) and the lower concentrations of serum IgG, IL-2, IL-10, IFN-γ (Figure 1) in model group mice showed that the immunosuppressed model was built successfully after being treated with 60 mg/kg bodyweight CP, compared with the normal group. As shown in Table 1, the spleen index was increased after being treated with 50, 100, and 200 mg/kg bodyweight CPP compared with model group (*p* < 0.05). The thymus and liver index in model mice did not show any difference compared to the mice in normal, but they increased significantly after treated with 200 mg/kg bodyweight CPP (*p* < 0.05), even higher than normal mice (*p* > 0.05) (Table 1). 

### 2.3. Effects of CPP on Serum Cytokines Secretion in CP-Treated Mice

To elaborate the immunomodulation of CPP, the secretions of serum cytokines were determined by enzyme linked immunosorbent assay (ELISA) kits. It can be seen from Figure 1 that the concentrations of serum IgG, IL-2, IL-10 and IFN-γ in CPP groups were significant higher than those in model groups (*p* < 0.01), approaching to the normal level, but did not show a dose-dependent increase. The immunomodulation effect of CPP was manifested according to these increasing secretions of cytokines and the effects of CPP on organ indices shown in Table 1. 

### 2.4. Effects of CPP on sIgA Secretion in CP-Treated Mice

**Figure 2:**
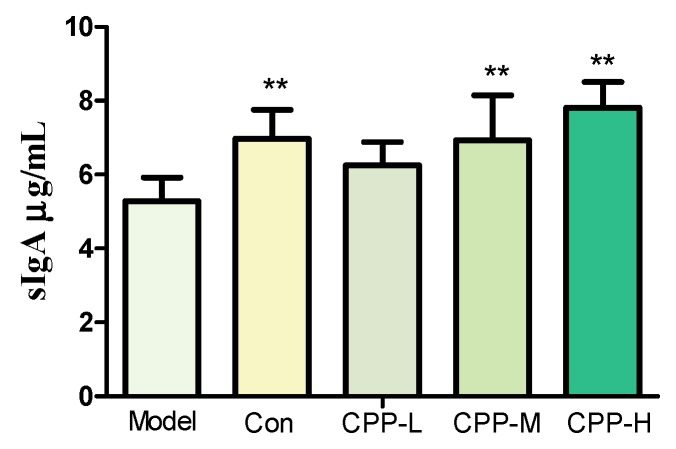
Effects of CPP on sIgA secretion in cyclophosphamide-treated mice. Model, model control; Con, normal control; CPP-L, 50 mg/kg bodyweight CPP treated group; CPP-M, 100 mg/kg bodyweight CPP treated group; CPP-H, 200 mg/kg bodyweight CPP treated group. The values were presented as mean ± SD. ** *p* < 0.01, compared with model group.

The sIgA, an immunoglobulin with antibacterial properties, is released in the intestinal lumen and served as the first line of defense to protect the intestinal epithelium from enteric toxins and pathogenic microorganisms [33,34]. The secretion of sIgA of ileum tissues was determined by ELISA kit after being homogenized with germ-free PBS to 50 mg/mL. The results have shown that the level of sIgA was decreased after intraperitoneal injection with CP (*p* < 0.01) (Figure 2), showed that the mucosal immunity was influenced. Meanwhile, the impaired intestinal mucosal function was resumed after taking 100 and 200 mg/kg bodyweight CPP (*p* < 0.01), indicating that the CPP could modulate the intestinal mucosal immune, like stimulating the secretion of sIgA.

### 2.5. Effects of CPP on the Number of Escherichia coli and Lactobacillus in CP-Treated Mice

The suspensions of cecum contents were inoculated in both MRS and MacConkey agar plate. Then, the number of *Lactobacillus* and *E. coli* were calculated three times. The results showed that the number of *Lactobacillus* was reduced (*p* < 0.01) and the *E. coli* was increased (*p* < 0.01) in CP-treated mice, indicating that there was probably a disturbance of intestinal flora. The CPP could regulate the unbalance by promoting the growth of *Lactobacillus*, and restrained the growth of *E. coli* (Table 2).

### 2.6. Effect of CPP on SCFA in CP-Treated Mice

**Figure 3:**
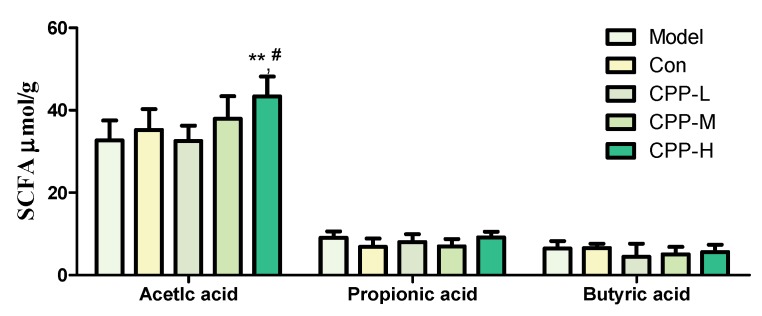
Effects of CPP on SCFA level in cyclophosphamide-treated mice. Model, model control; Con, normal control; CPP-L, 50 mg/kg bodyweight CPP treated group; CPP-M, 100 mg/kg bodyweight CPP treated group; CPP-H, 200 mg/kg bodyweight CPP treated group. The values were presented as mean ± SD. ** *p* < 0.01, compared with model group; ^#^ *p* < 0.05, compared with normal group.

SCFA mainly consist of acetic acid, propionic acid and butyric acid, are products of the fermentation of dietary fibers by the anaerobic intestinal beneficial bacteria, and have improvement on the host energy metabolism and inflammatory responses, because the host SCFA receptors and target molecules are expressed in both metabolic and immune tissues [35,36,37,38]. As shown in Figure 3, the CP did not decrease the acetic, propionic and butyric acid in cecum (*p* > 0.05). Meanwhile, the concentration of acetic acid in CPP-H group was higher than model group (*p* < 0.01) and normal group (*p* < 0.05), with a dosage-dependent manner. The results listed above indicated that the CPP could be fermented to acetic acid by the microbiota. 

## 3. Discussion

Many polysaccharides isolated from Chinese Traditional Medicines have been studied in the biomedical area and represented an activity for their immunostimulation on immune organs, cells, and genes, as potential immunological drugs [39]. There are numerous studies indicating that the stimulator effects of polysaccharides begin with the enhancement of the phagocytosis of macrophages and antigen processing capacity [1,40,41,42]. Then the acquired immunity was improved by cytokines secretion receptors, such as Dectin-1, Mannose receptor (MR), complement receptor 3 (CR3), toll-like receptors (TLR) and scavenger receptors (SR) [41]. 

CP is a potent immunosuppressive agent due to the repression of the production of immune-related cytokines (TNF-α, IFN-γ, IL-1α, IL-2, IL-6, etc.) and the activities of the natural killer cell (NK), lymphocyte in spleen, total white blood cells, platelet count, etc. [1,6,42,43]. It is well known that B lymphocyte is very susceptible to the suppressive action of CP, explaining that it had little effect on the thymus, the organ that T lymphocyte developed, differentiated and matured [44,45] in our study. Meanwhile, not only the spleen index, but also the thymus and liver index in CPP groups were increased compared to the model group, indicating that CPP was a potent immune-enhancer in accordance with the studies about polysaccharides from *C. pilosula* before [30,31,46].

The mechanisms of CP to influence the immune system were the Th2/Th1 differentiation, the induction of Th17 cells, the inhibition of regulatory T-cell (Treg) and the enhancement of T/B-cell proliferation and survival [42,47,48,49]. T-helper cells help B cells to divide, differentiate and make antibodies, generating their effects by releasing soluble cytokines. IL-2 could maintain T-cell proliferation, including promoting T-cell activation, differentiation and B-cell proliferation. It plays a key role in activation of cytotoxic T-cell (CTL) and NK cells as well, which are the two main cytotoxic lymphocytes to protect against tumor cells and viruses. Meanwhile, IL-2 modulates the expression of receptors for other cytokines and transcription factors, promoting or inhibiting cytokine secretion [42,50]. IFN-γ is produced by the CTL, NK cells, CD4^+^ T and CD8^+^ T cells. It is crucial for protection against viral, intracellular bacterial infections and tumor control, that the innate recognition of pathogens leads to the production of IFN-γ by NK and/or natural killer T (NKT) cells, which in turn influences the generation of IFN-γ-producing CD4^+^ and CD8^+^ T cells [42,51]. IL-10, a potent anti-inflammatory cytokine, is produced by many cells of the adaptive immune system, including Th1, Th2 and Th17 cell subsets, Treg cells, CD8+ T cells and B cells. It has mast cell growth factor activity in combination with IL-3 and/or IL-4, and T-cell growth factor activity on mature and immature mouse thymocytes in the presence of IL-2 and IL-4 [52,53]. These results suggested that CPP could restore the reduced cytokines of IL-2, IL-10, IFN-γ by CP (compared with model group), suggesting that it could probably modulate the differentiation of T lymphocyte in the humoral immunity, which resulted in the stimulation of cytokines secretion, similarly with the effects of polysaccharides from *Lycium ruthenicum* Murr [54], *Ganoderma atrum* [55], etc. At the same time, the IgG level and spleen index were restored by CPP. It was probable that CPP improved the growth of B lymphocytes in spleen or the differentiation into plasma cells by activating B-cell membrane receptors CD19, CD79b or TLR2/4, and then showed an increase in the secretion of immunoglobulin [39]. 

The human gastrointestinal (GI) tract carries more than 10^14^ microbial cells with more than a thousand diverse types, mainly comprising *Firmicutes*, *Bacteroidetes*, *Actino bacteria*, and *Proteobacteria,* residing at mucosal surfaces of the gastrointestinal tract, and is varied by diet, disease, environment, etc. [16]. It lives in a mutualistic relationship with the host, and plays an important role in the development of the immune system by activating the gut-associated lymph tissues (GALT), and regulating the secretion of sIgA, nutrients absorption, etc. [9,56,57]. In addition, the bacterial fermentation metabolite of fiber, such as SCFA, are beneficial to the host and associated with a reduced risk of different diseases, including IBD [13,35,36,37,56]. CP could increase the intestinal permeability and potentially pathogenic bacteria counts, such as *Escherichia coli*, *Pseudomonas* [7], and induces a reduction of bacteria specifics of the *Firmicutes* phylum, such as *Lactobacillus* in the intestinal mucosa [5]. Besides, the production of sIgA is dependent on the peyer’s patch M-cells, and processing by antigen-presenting cells such as dendritic cells (DCs), T-cell activation, and ultimately B-cell class switch recombination in GALTs [39], and sIgA secretion in intestinal mucosa was reduced by CP [58,59,60]. In our results, the decrease of the number of *Lactobacillus* in model group represented the inhibition effect by CP, similar to previous studies [23,24]. Combing our previous work [32], it was probable that the inulin composition of CPP restored the number of *Lactobacillus* in all CPP groups. Meanwhile, the increase in the number of *E. coli* was possibly a result of probably disrupted balance in intestinal micro-ecology by CP [7], as it showed an opposite trend with the number of *Lactobacillus*. All these results showed a protection of CPP from mucosal immune barrier disruption and a potential maintaining function of intestinal flora by stimulation growth of *Lactobacillus*, given a lower risk of inflammatory disease. Moreover, it was probable that most of the carbohydrate-degradation bacteria were not interfered by CP, because concentration of SCFA, the fermentation metabolite of polysaccharides, did not decrease in model group. However, there was a dose-dependent manner in CPP groups, in accordance with previous several studies that a diet with a high content of non-digestible fiber leads to higher levels of SCFAs in the intestine [61].

It is important that the structure and activities of CPP were analyzed in our previous studies, including the complement fixing activity of pectic polysaccharides composition [31], and the potential prebiotic activity of inulin-type fructan composition [32]. All these studies were established in vitro, and it was hard to explain clearly the active mechanism and connection between immune enhancement and intestinal flora regulation activity. In this study, we indicated that CPP could ameliorate the disorder of intestinal flora and strengthen the immune function at the same time, especially in mucosa immunity, the essential connection site of gut microbiota and immunity. Meanwhile, it was unclear which one is acting on the mucosal immune of CPP. Maybe it was the fermented product of polysaccharides, or the changed intestinal microbiota composition. It may also be the direct activation of certain relevant receptors in mucosa. More studies are needed to reveal the relationships between the intestinal microbiota and immunity.

## 4. Materials and Methods

### 4.1. Materials and Chemicals

The roots of *C. pilosula* Nannf. var. *modesta* L. T. Shen were collected in October 2016 from Jiuzhaigou County (Tibetan Qiang Autonomous Prefecture of Ngawa, China), and identified by Yuan-feng Zou, College of Veterinary Medicine, Sichuan Agricultural University. The roots were dried and pulverized to a fine powder.

The CP (C8650) was obtained from Solarbio technology Co., Ltd., (Beijing, China). The ELISA kits (including mouse IgG, sIgA, IL-2, IL-10 and IFN-γ) were purchased from Enzyme-linked Biotechnology Co., Ltd., (Shanghai, China). The MRS (HB0384) and MacConkey Agar (HB6238) were obtained from Hopebio Co., Ltd., (Qingdao, China). The d-glucose (47829), acetic acid (71251), propionic acid (94425) and butyric acid (19215) were purchased from Sigma-Aldrich (St. Louis, MO, USA); Folin-Cioalteu reagent (PRLA09050) was obtained from LI-DA Biotechnology Co., Ltd., (Shanghai, China); the gallic acid (98%, A51523) was purchased from Xiya Chemical Industries Co., Ltd., (Linshu, China). The bovine serum albumin (BSA, A8020) and coomassie brilliant blue G-250 (C8420) were purchased from Solarbio Science and Technology Ltd. (Beijing, China). All other chemicals, such as phenol, sulfuric acid, acetone, boric acid, glycerin, etc., were of analytical grade, obtained from the Chengdu Kelong chemical factory (Chengdu, China).

### 4.2. Preparation of Polysaccharides from C. pilosula 

The roots (200 g) were weighed and dried in air drying oven at 50 °C (183.54 g), and pulverized to a fine powder by a mechanical grinder, then passed through 0.25 mm mesh. It was extracted with refluxing 96% ethanol to remove low molecular weight and lipophilic compounds. The dried residual (175.80 g) were extracted twice with boiling water, 2.5 h each time, with solvent-material ratio of 40 mL/g. The crude extracts were concentrated (RE-3000, Yarong Biochemical Instruments Factory, Shanghai, China), then precipitated by 96% ethanol with 4-folds volume of concentrated extracts at 4 °C for 12 h. After centrifuged at 2129× *g* for 10 min, the precipitation was lyophilized, denominated as CPP [32].

### 4.3. Determination of Total Sugar, Polyphenol and Protein Content

The content of total sugar of CPP was determined by phenol-sulfuric acid method using d-glucose as standard [62]. The polyphenol and protein content were determined with the Folin-Ciocalteu assay [63] and the Bio-Rad protein assay, based on the method of Bradford [64], and the gallic acid (98%, in a concentration range of 6.8–61.2 μg/mL) and BSA (in a concentration range of 10–90 μg/mL) were used as a standard, respectively.

### 4.4. Animals and Experimental Design

All animal procedures were reviewed and approved by the Animal Care and Use Committee of Sichuan Agricultural University (2017-0608). Forty SPF BALB/c mice (8-weeks old, 18–20 g) were purchased from Dashuo Biotechnology Co., Ltd., (Chengdu, China). The animals were provided with water and mouse chow *ad libitum*, and were maintained in a specific pathogen-free environment, with a 12 h light-dark cycle and 22 ± 1 °C room temperature. After acclimatization under free access to food and water for 7 days, they were randomly divided into 5 groups (8 mice each group) as follows. The Group I was normal control group (Con), the Group II was model group (Model), and the Group III-V were supplemented of different dose of CPP. All the mice were treated saline or CP (60 mg/kg/d, 0.1 mL/10 g body weight) by intraperitoneal injection from days 1–3, and with saline or CPP by intragastric administrated (0.1 mL/10 g body weight) from days 4–10 (Table 3) [43]. 

Twenty-four hours after the last drug administration, the animals were weighed and then sacrificed by decapitation. The spleen, thymus and liver, were exercised from the animals, and were weighed immediately to calculate the index according to the following formula: index (mg/g) = (weight of organ/body weight). The blood, cecum content and the ileum tissues were obtained from all mice for the following studies.

### 4.5. Determination of IgG, IL-2, IL-10, IFN-γ in Serum

The blood samples were collected by excising eyeball after anesthesia, and then the serum was obtained after coagulated at room temperature and separated by centrifuging at 2862× *g* for 15 min. The IgG, IL-2, IL-10, IFN-γ content in the serum were determined according to the manufacturer’s instruction. 

### 4.6. Determination of sIgA in Ileum Tissues

The ileum tissues were obtained after dissection and stored at −80 °C. They were ground using liquid nitrogen in sterile mortars, then weighed and homogenized with germ-free PBS to 50 mg/mL. The supernatants were collected after centrifuged at 4 °C, 2862× *g*, removing the tissue fragments, and then the concentration of sIgA was determined using ELISA kit.

### 4.7. The Bacteriologic Analysis 

The fresh cecum content were collected on the last day in the experiment and divided into two groups stored at 4 °C and −20 °C for the determination of the number of *Lactobacilli*, *Escherichia coli* and the SCFA content, respectively. 

The cecum contents were weighted and homogenized in saline (10 mg/mL), and repeatedly diluted in 10-fold step from 10^3^–10^5^ for bacteria counts. Each dilute (50 μL) was transferred to the corresponding selective culture medium for aerobe (DHP-9082, Jiecheng Experimental Apparatus Co., Ltd., Shanghai, China) and anaerobe (Thermo Scientific 1029, Waltham, Massachusetts, USA, in 85% N_2_, 10% H_2_, 5% CO_2_) cultivation [7]. The MRS medium was used for *Lactobacillus* counts, and the MacCon Agar was used for *E. coli* counts. The bacteria count was expressed as log^10^ CFU (colony-forming unit)/g of cecum content) and was identified to the level of genus. Each dilute was set in triplicate above. 

### 4.8. Determination of SCFA in Cecum Content 

The cecum contents were obtained and stored according to 4.7, then weighed and homogenized in 150 μL ddH_2_O, placed for 30 min in room temperature. The supernatant (100 μL) was collected after centrifuged at 9600× *g*, 10 min, then adding 20 μL 25% metaphosphoric acid, 1.52 μL 210 mmol/L crotonic acid (internal standard), and placed at 4 °C for 30 min. After centrifuged at 13,800× *g* 10 min, the supernatant was mixed with ethanol (1:1) and filtered through 0.22 μm filter membrane for gas chromatography (GC) determination (X). The concentration (M, mmol/L) of acetic acid, propionic acid and butyric acid was calculated according to the following formula:

(1) M=X×2×1.2152

The standard curve was established by the standard acetic acid, propionic acid and butyric acid with the same method above [65,66], with detection threshold of 0.901~4.503 mmol/L, 0.838~4.190 mmol/L, and 0.273~1.363 mmol/L, respectively.

The samples were analyzed using a Varian CP-3800 (Palo Alto, CA, USA) with flame ionization detector (FID), attached to a HP-FFAP column (30 m × 0.535 mm × 1 μm). The temperature of injection and detector were 220 °C and 250 °C, respectively. The column temperature was 100 °C when the sample was injected, and then increased with 20 °C/min to 190 °C, and then keeping 0.5 min. Nitrogen was the carrier gas, at speed of 35 mL/min and split ratio of 100:1. The injection volume was 1 μL [65,66,67].

### 4.9. Statistical Analysis

The statistical values were represented as mean ± SD, and the statistical comparisons were applied with the one-way analysis of variance (ANOVA) by Duncan’s test using SPSS version 20.0 (IBM, Armonk, NY, USA), then the values of *p* < 0.05 and *p* < 0.01 were considered statistically significant and highly significant, respectively.

## 5. Conclusions

The polysaccharides from *C. pilosula* roots have been shown to have good immune enhancement in previous studies, both in vitro and in vivo. However, they did not show any possible mechanism. The polysaccharides from *C. pilosula* Nannf. var. *modesta* (Nannf.) L. T. Shen (CPP) showed immunomodulatory effects, including promoting the secretion of IgG and cytokines (IL-2, IL-10, IFN-γ) in serum. More importantly, the intestinal mucosal immunity was increased in immunosuppressed mice by stimulating the secretion of sIgA. At the same time, the impaired intestinal flora by facilitating the growth of *Lactobacillus* was restored by CPP, and metabolism (acetate acid) production of it was increased in our study. Considering the metabolic characteristics of polysaccharides and the composition of CPP (the immunomodulatory pectic polysaccharides and prebiotic inulin-type fructan), we inferred that the intestine mucosa or microbiota was the potential target active site. In future work, we will explore the detailed mechanism in vivo, such as fecal microbial transplantation or gene-deficient mice.

## Figures and Tables

**Table 1:** Effects of CPP on spleen, thymus and liver index in cyclophosphamide-treated mice.

Group ^a^	Spleen Index	Thymus Index	Liver Index
Model	4.71 ± 0.75	1.87 ± 0.27	57.93 ± 0.51
Con	7.72 ± 1.75 *	1.76 ± 0.21	55.98 ± 1.36
CPP-L	6.71 ± 3.64 *	1.46 ± 0.33	57.35 ± 4.15
CPP-M	11.16 ± 1.05 **#	1.87 ± 0.49	62.89 ± 2.58 **#
CPP-H	9.08 ± 0.41 **	2.24 ± 0.41 #	61.96 ± 0.70 *#

^a^ Model, model control; Con, normal control; CPP-L, 50 mg/kg bodyweight CPP treated group; CPP-M, 100 mg/kg bodyweight CPP treated group; CPP-H, 200 mg/kg bodyweight CPP treated group. The values were presented as mean ± SD. * *p* < 0.05, compared with model group; ** *p* < 0.01, compared with model group; ^#^ *p* < 0.05, compared with normal group.

**Table 2:** Effects of CPP on the number of *E. coli* and *Lactobacillus* in cyclophosphamide-treated mice.

Group ^a^	*E. coli*/log^10^ CFU/g	*Lactobacillus*/log^10^ CFU/g
Model	6.69 ± 0.61	10.98 ± 0.33
Con	5.52 ± 0.41 **	11.69 ± 0.17 **
CPP-L	6.77 ± 0.32	11.16 ± 0.39
CPP-M	5.97 ± 0.53 *	11.23 ± 0.44
CPP-H	6.56 ± 0.68	11.78 ± 0.07 **

^a^ Model, model control; Con, normal control; CPP-L, 50 mg/kg bodyweight CPP treated group; CPP-M, 100 mg/kg bodyweight CPP treated group; CPP-H, 200 mg/kg bodyweight CPP treated group. The values were presented as mean ± SD. * *p* < 0.05, compared with model group; ** *p* < 0.01, compared with model group.

**Table 3:** The details of five groups.

Group	Days 1–3	Days 4–10
Group I (Con)	Saline	Saline
Group II (Model)	CP	Saline
Group III (CPP-L)	CP	50 mg/kg CPP BW/d
Group IV (CPP-M)	CP	100 mg/kg CPP BW/d
Group V (CPP-H)	CP	200 mg/kg CPP BW/d
